# Effect of Acetaminophen (APAP) on Physiological Indicators in *Lactuca sativa*

**DOI:** 10.3390/life10110303

**Published:** 2020-11-23

**Authors:** Jiri Kudrna, Frantisek Hnilicka, Jan Kubes, Pavla Vachova, Helena Hnilickova, Margita Kuklova

**Affiliations:** 1 Department of Botany and Plant Physiology, Czech University of Life Sciences Prague, 165 00 Prague, Czech Republic; kudrnaj@af.czu.cz (J.K.); kubes@af.czu.cz (J.K.); vachovap@af.czu.cz (P.V.); hnilickova@af.czu.cz (H.H.); 2 Institute of Forest Ecology of the Slovak Academy of Sciences, 960 53 Zvolen, Slovakia; kuklova@ife.sk

**Keywords:** *Lactuca sativa*, acetaminophen, APAP, photosynthesis, fluorescence

## Abstract

This study analyzes the effects of acetaminophen (APAP) as a contaminant on physiological characteristics of lettuce plants (*Lactuca sativa* L.). Experiments were provided in an experimental greenhouse with semi-controlled conditions. The effect of different amounts of contaminant was evaluated by using regression analysis. Plants were grown in five concentrations of APAP: 0 µM, 5 µM, 50 µM, 500 µM, and 5 mM for 14 days in two variants, acute and chronic. The obtained results show that the monitored parameters were demonstrably influenced by the experimental variant. Plants are more sensitive to chronic contamination compared to acute. Significant (*p* < 0.05) deviation in photosynthesis and fluorescence was observed compared to the control in different variants. The highest doses of APAP reduced the intensity of photosynthesis by a maximum of more than 31% compared to the control. A reduction of 18% was observed for the fluorescence parameters. Pronounced correlation was described between chlorophyll fluorescence parameters and yield mainly under APAP conditions. The amount of chlorophyll was influenced by exposure to APAP.

## 1. Introduction

With the growth of the human population and the development of the pharmaceutical industry, natural resources are becoming more contaminated, which in turn affect the entire food chain. These are, for example, the pollution of drinking water, agricultural land, and food by pharmaceuticals [1]. According to the European Commission (Green Deal), a significant amount of total chemical production can have harmful effects on the environment. Global drug production and subsequent water contamination can pose a threat to human health, as many of these substances and their subsequent metabolites can affect not only us [2,3], but also ecosystem dynamics [4]. Contamination of the aquatic environment with pharmaceutical compounds occurs mainly as a result of pharmaceutical production, waste policy of hospital facilities, inefficient waste disposal, or normal consumer use [5]. Examples of real contamination were already described across Europe [6,7,8]. Today’s commonly used water purification processes cannot effectively remove pharmaceutical residues, and it is therefore necessary to focus on this issue [9,10]. Acetaminophen (APAP) is one of the world’s most widely used drugs (non-opioid analgesic and antipyretic agents) available without a prescription [11]. Studies on the use of sewage sludge for fertilizing fields and crops have been the subject of research for several decades [12,13]. The presence of drugs has been demonstrated in sewage sludge several times [14,15,16]. Commonly detectable concentrations of APAP in the environment and in water after treatment with water treatment plants range from 1–6 μg/L and up to 10 μg/L, respectively [17].

While the effect of pharmaceutical residues is known in animals’ metabolism [18,19], in the case of plants, the toxicological significance is less studied. Xenobiotic substances can be accumulated in plants in various ways and potentially contaminate the food chain [20,21,22]. As part of the study of the effect of drugs and their metabolites, the primary metabolism, electron transport, was studied in plants. Experiments with wheat (*Triticum aestivum* L.) resulted in changes in photosynthetic pigments and inhibition of seedling growth [23]. An investigation of APAP and the diclofenac effect on *Allium cepa, Lactuca sativa, Pisum sativum, Solanum lycopersicum*, and *Zea mays* plants was provided, and in maize plants, for example, a 40% decrease in the reduction in quantum yields of photosystem II was measured at a dose of 10 mg/L of both chemicals [24]. In the metabolism of horseradish (*Armoracia rusticana* L.) under APAP stress plants subsequently received and degraded it as similar xenobiotics [25]. This statement can be taken as supporting fact for concerns about the possible presence of APAP and its metabolites in plants and products for human consumption. Due to the possible incorporation of APAP into the structures of the plant body, we set the following goal for this experiment: The aim of this experiment was to study the effect of increasing concentrations of APAP on primary metabolism and chlorophyll fluorescence as relevant characteristics of the state of juvenile lettuce plants (*Lactuca sativa* L.) (plant condition). The impact of stress effect depends on the amount and severity of the stressor. Low stress levels might not affect the efficiency of photosystem II (PSII) in a direct way but can cause side symptoms of stress such as stomatal closure, for example, in the case of drought stress, which leads to decreasing CO_2_ assimilation and later ATP metabolism defects. It implies the need for thorough and comprehensive research [26]. The idea that chlorophyll fluorescence could be quenchable by stress was already mentioned [27] in connection with decreasing PSII quantum yield and electron transport rates due to drought stress in *Phaseolus vulgaris* plants.

Based on these literature data, the objectives of the work were to determine whether paracetamol concentrations affect the primary metabolism of juvenile lettuce plants and whether the plants respond differently to a single application or to the chronic effects of APAP.

## 2. Materials and Methods

### 2.1. Plant Material and Experimental Conditions

The seeds of *Lactuca sativa* L. var. capitata “Král Máje 1” were obtained from SEMO a.s. The plants were pre-grown in a hydroponic system in rockwool planting blocks. The experiment was provided in partially controlled greenhouse conditions at the Czech University of Life Sciences Prague, Czech Republic. The greenhouse is located at GPS: 50.129976, 14.373707. The experiments took place on two dates in spring and autumn 2019. The experiment was conducted under semi-controlled conditions (natural light conditions, air temperature 25 ± 2/20 ± 2 °C day/night, relative air humidity 65% min and 85% max). The light regime was natural without shading the greenhouse (14/10 h). The experimental plants were grown in containers with a volume of 5 dm^3^ in the garden substrate (AGRO CS: pH 5.0–6.5, nutrient content 100 mg N/L, 44 mg P/L, 124 mg K/L).

The experiment was based on two basic variants: a single application of APAP (acute toxicity) and chronic (multiple) exposure; see Table 1. The amount of irrigation water was 320 mL of SDV (sterilized distilled water) with the appropriate amount of APAP every other day. Acetaminophen (APAP), chem.: N-acetyl-para-aminophenol (reference standard) with a guaranteed purity of 99.9%, was purchased from Sigma-Aldrich^®^ (Sigma-Aldrich, Steinheim am Albuch, Germany). The dissolution of the APAP in SDV was performed using an ultrasonic homogenizer. The following concentrations of APAP—0 (control), 5 µM, 50 µM, 500 µM, and 5 mM solutions—were used during the experiment.

### 2.2. Plant Analyses

Measurements of the physiological characteristics were performed at intervals of 0 h, 24 h, 72 h, 168 h, 240 h, and 360 h after the first application of acetaminophen (APAP). The pigment content in the leaves was measured photometrically with an Evolution 2000 UV-Vis (Thermo Fisher Scientific Inc., Waltham, MA, USA) by the Porra method [28]. The rate of photosynthesis (P_n_) was measured on the upper surface of the leaves (the middle part of the leaf blade) in situ using the portable gas exchange system LCpro+ (ADC BioScientific Ltd., Hoddesdon, UK). P_n_ was measured under adjusted light and temperature conditions. The irradiance was 650 µmol/m^2^/s of photosynthetically active radiation (PAR) and the temperature in the chamber was 25 °C. The minimum chlorophyll (Chl) *a* fluorescence (F_0_) and the maximum Chl *a* fluorescence (F_m_) were also measured in situ with the portable fluorometer OSI 1 FL (ADC BioScientific Ltd., Hoddesdon, UK) with 1 second excitation pulse (660 nm) and saturation intensity of 3000 μmol/m^2^/s after 20 min of dark adaptation of the leaves. The maximum quantum efficiency of photosystem II (F_v_/F_m_) was calculated as F_v_/F_m_ (F_v_ = F_m_ − F_0_). Chlorophyll fluorescence is a common physiological parameter for studying plant stress in physiology research [29,30,31].

### 2.3. Data Analysis

The data were analyzed with the use of the Statistica 12 software (StatSoft Ltd., Tulsa, OK, USA). The variability of the measured characteristics between the studied plots was tested by a one-way ANOVA model (α = 0.05) and a Fisher LSD test. The impact of APAP on physiological parameters was analyzed with the regression method, using polynomial functions. For the statistical analysis of the results five replicates were used.

## 3. Results and Discussion

The effect of the acute and chronic effects of paracetamol on the content of photosynthetically active pigments, the rate of photosynthesis, and the chlorophyll fluorescence of juvenile lettuce plants was observed. Stress factors cause numerous changes in several physiological characteristics, e.g., gas exchange, photosynthetic content of active pigments, dry matter formation, plant growth, etc. [25,32]. Chlorophyll fluorescence is considered a useful tool for stress response determination [33]. There is a possibility that PSII can be damaged by stress, which can escalate to lower electron transfer and prolonged time before reaching maximum fluorescence intensity [34]. The rate of photosynthesis is also considered a relevant parameter while analyzing levels of stress in plants [35].

### 3.1. Acute Exposure to APAP

It can be seen from the Table 2 that significant differences were found between the individual concentrations of APAP in the solutions and applications. Significant differences in the content of total chlorophyll and carotenoids in the leaves were found, for example, in the second highest concentration of APAP (500 μM/L) in the chronic action. This difference is evident between acute and chronic effects, but also within individual concentrations. The content of chlorophylls and carotenoids in the leaves of the control plants was inconclusively lowest in comparison with the treated variants (3.495 nM/cm^2^ and 0.581 nM/cm^2^). Due to the delivery of APAP to the solution, there was an inconclusive increase in the content of pigments in the leaves during the acute action of said xenobiotic. At the highest concentration of APAP in solution, the amount of chlorophyll in the leaves increased by 8.12% and carotenoids by 21.51% compared to the control plants. In the case of chronic treatment, a statistically significant increase in the content of photosynthetically active pigments was found in the variant with a concentration of APAP of 500 μM by 27.46% (chlorophyll) and 41.8% (carotenoids).

Our results show that increasing concentrations of APAP also increase the content of total chlorophyll, which was confirmed, for example, in [36]. The authors studied the effect of diclofenac on tomatoes. On the other hand, the conclusions of, e.g., [37], in the case of *Lemna minor* and *Phaseolus vulgaris* [38], suggest that the effect of APAP on plant photosynthetic processes may be species dependent. In accordance with the work of [35], and increased content of chlorophyll *b* appears to be an adaptive mechanism to the toxic effects of diclofenac. According to [39], an increase in chlorophyll content may be related to a decrease in the enzymatic activities of nicotinamide adenine dinucleotide phosphatedependent Chl (ide) b reductase and ferredoxin-dependent hydroxymethyl Chl(ide) reductase, but further studies are needed to clarify this mechanism. For the study of biosynthetic pathways and enzymatic activity, the content of carotenoids in the leaves increased with increasing concentration of APAP in solution. The results correspond to the conclusions presented, for example, in *Lemna minor* and *Lemna gibba* [40] after diclofenac treatment. In accordance with the conclusions in [40,41], it can be stated that increased levels of carotenoids in leaves is a response of plants to xenobiotics and thus provide protection against oxidative damage. Another role of carotenoids is the protection of reaction centers and the antenna complex against oxidative stress.

**Figure 1:**
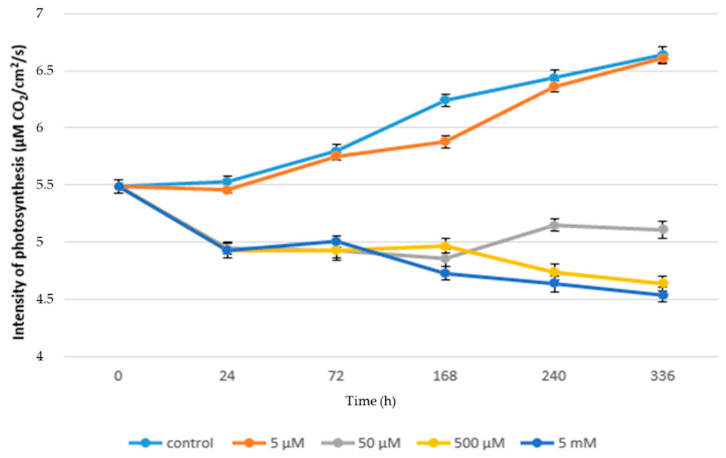
Rate of photosynthesis (µM CO_2_/cm^2^/s) in acute toxicity.

Figure 1 shows the negative effect of paracetamol on the rate of photosynthesis of juvenile lettuce plants, where in the case of plants treated with paracetamol the rate of photosynthesis ranged from 5.49 to 4.54 µM CO_2_/cm^2^/s. While in the control plants the range of photosynthesis values was 5.49 to 6.64 µM CO_2_/cm^2^/s, in control plants, the rate of photosynthesis was significantly increased over time from 72 h to 336 h. The increase was 14.48%. A similar trend can also be observed in lettuce plants grown in the lowest concentration of paracetamol (5 µM). In these plants, the rate of photosynthesis was from 5.49 to 6.61 µM CO_2_/cm^2^/s. Compared to the control plants, there was an inconclusive reduction of photosynthesis rate by 0.45% in the final phase of the experiment. Our results show that low concentrations of APAP do not inhibit (or negligibly inhibit) the rate of photosynthesis. Similar results after the application of low concentrations of APAP were observed in duckweed (*Lemna minor*) [42]. This effect is probably related to the fact that these low concentrations of APAP do not have a destructive effect on the photosynthetic apparatus of plants. This is mainly due to the low negative effect on gas exchange parameters, including ribulose-1,6-bisphosphate carboxylase/oxygenase (RUBISCO) enzyme activity, the number of vents, and electron transfer within PSII.

With increasing concentrations of paracetamol, the inhibitory effect of its higher concentrations on the rate of photosynthesis was demonstrated. As can be seen from Figure 1, the highest concentration of paracetamol significantly reduced the rate of photosynthesis of plants in comparison not only with control plants, but also with other treated variants. In plants treated with the highest concentration of paracetamol, the rate of photosynthesis was significantly reduced to 4.93 µM CO_2_/cm^2^/s within 24 h of application. Thereafter, photosynthesis increased inconclusively, but from 168 h from APAP application until the end of the experiment, a decrease in photosynthesis from 4.73 to 4.54 µM CO_2_/cm^2^/s was noted. In the case of a concentration of 500µM, the rate of photosynthesis from 24 h to 168 h was stable and a significant decrease in its value was found in the measurement terms of 240 h to 4.74 µM CO_2_/cm^2^/s and 336 h to 4.64 µM CO_2_/cm^2^/s. These results indicate that high concentrations of APAP have an inhibitory effect on the rate of photosynthesis. Similar results after the application of APAP were confirmed in bean plants (*Phaseolus vulgaris* L.) [43]. The reduction in the intensity of photosynthesis rates is firmly associated with changes in the content of photosynthetically active pigments, namely with an increased content of carotenoids. In addition, the increased peroxisome activity is mainly due to the strong activation of the photorespiratory pathway associated with the inhibition of photosynthesis. It can therefore be assumed that similar phenomena occurred in our experiments [44].

In contrast, the rate of photosynthesis increased in this time period in plants treated with concentrations of 50 µM from 4.86 µM CO_2_/cm^2^/s to 5.15 µM CO_2_/cm^2^/s (240 h) and 5.11 µM CO_2_/cm^2^/s (336 h), as shown in Figure 1. The highest final value of the decrease in photosynthesis intensity was observed in the variant treated with the highest concentration of APAP (5 mM) when there was a decrease in photosynthesis intensity by 31.62% in comparison with the control. Changes in the rate of photosynthesis and related parameters due to contamination of the external environment had already been observed [45,46,47,48]. Thus, the reduction in the rate of photosynthesis is probably due to the phytotoxicity of APAP, as a similar reaction was observed with diclofenac. Furthermore, it is possible that inhibition of photosynthesis appears to be related to reduced activity of PSII reaction centers.

Furthermore, the chlorophyll fluorescence parameters were measured. Individual parameters are listed in Table 3, which shows that the paracetamol-treated plants show a statistically significant reduction in the values of the chlorophyll fluorescence parameters compared to the control plants. Electron transport within PSII is sensitive to changes of external environment e.g., contamination with hazardous substance or other stresses [49]. The reduction in photosynthetic processes that are associated with both photosystems is manifested, for example, in drought stress, salinity, heat stress, xenobiotics, and herbicidal stress, but also in biogenic stressors. The effect on chlorophyll fluorescence could also be related to the reduced rate of gas exchange [50]. In the case of the evaluation of the F_v_/F_m_ ratio, it can be stated that this parameter decreased statistically most significantly in the 5 mM variant (0.765) compared to the control plants and, conversely, the lowest decrease was recorded in the 5 µM variant (0.788). It is generally reported in the literature as a non-stress value of the F_v_/F_m_ ratio of 0.820. Therefore, the obtained results show that chlorophyll fluorescence is sensitive to environmental contamination. This is in accordance with [29,51,52]. Based on the obtained results and in accordance with the literature, it can be assumed that the limitation of fluorescence is due to changes in the properties of P700, which reduces the quantum yield (F_v_/F_m_). This phenomenon was not only found for drugs, but also, for example, for irradiation [53]. The effect of APAP on fluorescence characteristics is probably also related to its possible toxicity, especially at higher concentrations, as is the case with diclofenac [42].

A similar trend can also be found in the case of the F_v_/F_0_ ratio, where the control plants had a ratio of 4.814, and in the plants treated with 5 µM APAP 4.508 and 5 mM 4.179. In contrast, the F_m_/F_0_ ratio showed the lowest reduction compared to control plants in the 5 µM APAP variant (5.747) and, conversely, the largest reduction in the 50 µM concentration (5.197). Compared to the F_v_/F_m_ ratio, it is a more appropriate indicator of the measured effect of stress on plants to use the F_v_/F_0_ ratio. The results show a difference in this fluorescence parameter due to the different concentration of APAP in the substrate. The above conclusion is confirmed in [54,55].

Thus, it can be assumed that higher concentrations of APAP lead to photosystem II (PSII) being inactivated. This inactivation leads to an increase in the basic fluorescence of chlorophyll, which is reflected in the F_v_/F_0_ ratio. The decrease in the baseline fluorescence ratio by the action of a stressor is probably related to the action of oxidative stress induced by reactive oxygen species, which cause damage to the PSII reaction centers and an increase in non-photochemical quenching (NPQ) and a release of thermal energy.

**Figure 2:**
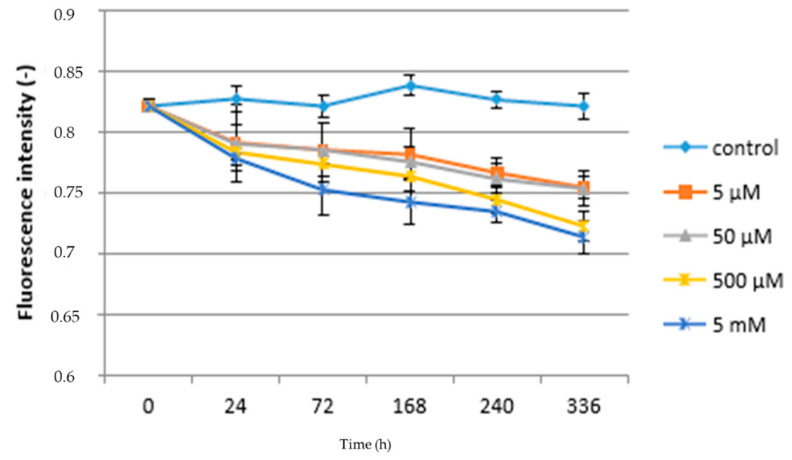
Fluorescence intensity—acute toxicity.

Figure 2 shows the relationship between paracetamol concentration, the duration of exposure, and the F_v_/F_m_ ratio. It follows that in the case of control plants, this characteristic was not significantly affected by the length of the experiment, when the lowest value of the F_v_/F_m_ ratio found at the beginning and end of the experiment was 0.822 and the highest was 168 h after the start of the experiment (0.839). In the case of plants grown at the lowest concentration of paracetamol (5 µM), the value of the F_v_/F_m_ ratio significantly decreased compared to the control during second day after application (24 h) of APAP (0.792) until the end of the experiment (0.755). This trend can be observed in all variants treated with APAP. A significant reduction in F_v_/F_m_ ratio values was found between the 5 µM APAP and 50 µM APAP variants, with an F_v_/F_m_ ratio interval for this variant from 0.754 (336 h) to 0.822 (beginning of experiment). In the case of the two high concentrations of paracetamol in solution, statistically significant differences to lower concentrations were already found, but also between each other. While at a concentration of 500 µM the lowest F_v_/F_m_ value was found 336 h after application at 0.723, at a concentration of 5 mM it was 0.714. Immediately at the beginning of the experiment (24 h after application), the F_v_/F_m_ ratio was 0.784 (500 µM) and 0.779 (5 mM); see Figure 2. Therefore, the obtained results show that chlorophyll fluorescence can be sensitive to environmental contamination. This is in accordance with the work mentioned above [29,51,52]. Pigments that are part of the photosynthesis process can reflect levels of sensitivity to stress conditions. In some cases, the amount of chlorophyll in plants can be highly volatile during stress response [56,57], because the content of photosynthetically active pigments is generally considered to be an indicator of a stress response. Changes in the pigment content can subsequently lead to a reduction in the ability to capture light, and thus affect photosynthesis and electron transfer within photosystem I (PSI) and photosystems II (PSII).

### 3.2. Chronic Exposure to APAP

**Figure 3:**
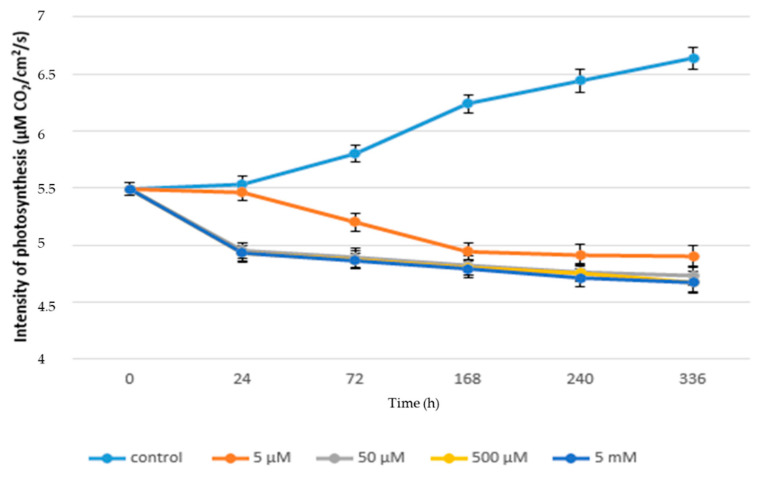
Rate of photosynthesis (µM CO_2_/cm^2^/s) in chronic toxicity.

The rate of photosynthesis of juvenile lettuce plants exposed to chronic paracetamol (excluding control) ranged from 5.46 to 4.67 µM CO_2_/cm^2^/s, as shown in Figure 3. The lowest average rate of photosynthesis was recorded for the 5 mM variant (4.67 µM CO_2_/cm^2^/s) compared to the control plants (6.64 µM CO_2_/cm^2^/s). Inconclusive differences in the rate of photosynthesis were found between the variants with 5 mM (4.93 µM CO_2_/cm^2^/s), 500 µM (4.93 µM CO_2_/cm^2^/s), and 50 µM (4.95 µM CO_2_/cm^2^/s). These results indicate that high concentrations of APAP have an inhibitory effect on the rate of photosynthesis, which is also in accordance with results of the experiments provided with duckweed (*Lemna minor*) [34]. These changes in higher concentrations of hazardous substances in the environment, including their long-term effect on the photosynthetic apparatus, lead to reduced photosynthesis due to inhibition of photosynthesis by affecting leaf chloroplast structure, light energy absorption, photosynthetic electron transport, stomatal conductivity, and Calvin cycle enzymatic activity exposure to heavy metals. This is in accordance with other studies [58].

Figure 3 shows a significant increase in the rate of photosynthesis as a function of time in the control plants, with a photosynthesis rate of 5.49 µM CO_2_/cm^2^/s at the beginning of the experiment and 6.64 µM CO_2_/cm^2^/s at the end of the experiment. Changes in the rate of photosynthesis as a function of time were already observed [59]. In plants treated with 5 µM of APAP, the rate of photosynthesis decreased compared to control plants from the second sampling (5.2 0 µM CO_2_/cm^2^/s) to the end of the experiment (4.9 µM CO_2_/cm^2^/s). From 168 h to 336 h after the beginning of the experiment, the decrease in photosynthesis was not statistically significant. In the case of plants treated with 50 µM, 500 µM, and 5 mM APAP, the trend of decreasing photosynthesis values was the same, with a decrease in photosynthesis from the start of the experiment from 5.49 to 4.73 µM CO_2_/cm^2^/s for the 50 µM variant, and from 5.49 to 4.67 µM CO_2_/cm^2^/s for the 500 µM and 5 mM variants, as shown in Figure 3. No significant differences were found between these (50 µM, 500 µM, 5 mM) variants.

The chronic effect of paracetamol significantly influenced the values of chlorophyll fluorescence, as shown in Table 3. It shows that all monitored parameters were significantly lower in plants treated with paracetamol compared to the control plants. Chronic exposure to pollutants and xenobiotics in comparison with the acute treatment significantly affects the level changes of electron transport, i.e., chlorophyll fluorescence parameters. This conclusion was confirmed in our experiments and is in line with [60], when the stress response is influenced by the duration of exposure to the stressor, but also depending on the concentration and developmental stage, when juvenile plants are usually more sensitive than older plants. Usually, the higher the strength of the stressor, the more the appropriate response of the plant increases. For the basic fluorescence parameter, the F_v_/F_m_ ratio, this value decreased by 9.81% (0.745) in the case of the highest paracetamol concentration (5 mM) compared to the control. On the other hand, the lowest reduction (5.21%) was found for the paracetamol concentration of 5 µM (0.783). The table also shows that in the case of the F_v_/F_0_ ratio the trend is the same as the previous measurement parameter, as the highest decrease in this indicator was recorded in the 5 mM APAP variant (3.921) and the lowest in the 5 µM variant (4.079), while the average value of the F_v_/F_0_ ratio of the control plants was 4.814. In the case of the F_m_/F_0_ characteristic, it was found that the most significant decrease compared to the control (5815) was determined at concentrations of 5 µM (5.208) and 5 mM (5.266); see Table 3.

**Figure 4:**
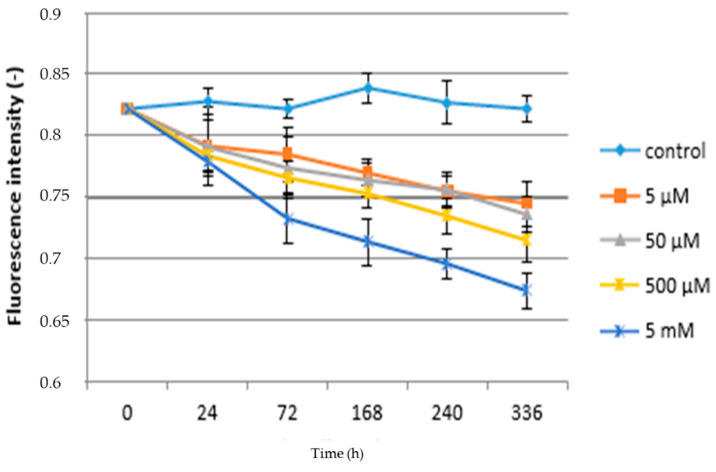
Fluorescence intensity—chronic toxicity.

Changes in F_v_/F_m_ values as a function of time and paracetamol concentration are shown in Figure 4. In both cases of chronic and acute treatment, it can be stated that the control variants have the highest values of selected physiological characteristics compared to plants treated with APAP. The results show a decrease in this fluorescence parameter due to the different concentration of APAP in the substrate. The above conclusions are consistent with [61,62]. Related issues are reported in [63], who studied the effect of heavy metals on the alga *Halophila ovalis*. It can be stated that the longer the exposure time and the concentration of xenobiotics, the higher the appropriate response of plants is to this fact. In the case of lettuce plants, an increase of F_0_ while F_m_ remains stable has also been shown; therefore, there is an overall decrease in the F_v_/F_m_ ratio and quantum yield. The increase in the F_0_ indicator is probably related to the action of APAP (and hazardous metals) on the PSII reaction center [64] or reducing energy transfer within light-harvesting pigments. Significant differences can be found between higher concentrations of paracetamol with each other and also in comparison to low concentrations. In the case of the lowest concentration (5 µM) of APAP the F_v_/F_0_ ratio decreased significantly 24 h (0.792) and kept decreasing until the end of the experiment after 336 h (0.745). Figure 4 further shows that all concentrations, in comparison with the control plants, reduced F_v_/F_m_ values during the time period. F_v_/F_m_ in plants grown in the highest concentration (5 mM) of APAP reached 0.674, which is more than an 18% decrease in comparison to the control plants. An analogous experiment with maize (*Zea mays*) observed a decrease in the F_v_/F_m_ ratio of up to 40% [24]. A decrease in the F_v_/F_m_ in willow plants (*Salix babylonica* L.) influenced by phenol contamination was close to our results [65]. A similar statement was made by [63], who studied the effect of heavy metals on chlorophyll fluorescence in *Halophila ovalis*. Based on the obtained results, it can be stated that the reduction of the quantum fluorescence yield and the F_v_/F_m_ ratio is related to changes in the ultrastructure of the thylakoid membrane, which would affect the electron transfer rate and further reduction of PSII photochemical efficiency probably related to antenna pigment destruction.

The results of the regression analysis show that there is a mean relationship between the rate of photosynthesis and the concentration of paracetamol. The calculated regression coefficient at the level of α = 0.05 and *p* = 0.00000 was 0.3920 and the equation y = 30.460 − 0.245c (y = Pn and c = APAP). The low regression coefficient for changes in the rate of photosynthesis depending on the action of the stressor may indicate that this parameter is not very sensitive to the detection of the stress response, as also stated [66] in experiments with rice (*Oryza sativa*). Based on the regression analysis, it can be concluded that there is no close dependence between chronic and acute drug action on changes in the rate of photosynthesis. The calculated coefficient was 13.83%; therefore, it can be stated that photosynthesis is affected polyfactorially. Polyfactorial effects on the rate of photosynthesis are also confirmed by [67] in sugar beet plants during stress. In the case of the effect of different paracetamol concentrations on chlorophyll fluorescence, it can be stated that the dependence between APAP concentration and fluorescence is weak, as the coefficient is 0.2874. The proposed equation would be in the form of y = 2.418 – 0.016c (y = fluorescence and c = APAP). Consistent with the rate of photosynthesis, no close dependence was found between the chronic and acute effects of paracetamol on chlorophyll fluorescence, as the calculated regression coefficient was 0.65. The relatively high regression coefficient shows that the fluorescence parameters appear to be a suitable indicator of the degree of stressor effect. This conclusion is also confirmed by [68] in barley (*Hordeum vulgare*) plants. The relationship between the rate of photosynthesis and chlorophyll fluorescence can be expressed by the equation y = 4.560 + 1.110 F_v_/F_m_, while based on the calculation of the regression coefficient it can be stated that this is not a close dependence, as the dependence is low (17.25%). The relationship between photosynthesis and fluorescence is mentioned in [69] and describes deeper connections between them.

## 4. Conclusions

The results showed that the observed physiological characteristics were influenced by different amounts of applied APAP. Excessive amounts of APAP caused significant decrease in the rate of photosynthesis as well as chlorophyll *a* fluorescence, which shows a possible effect of the quantum efficiency of photosystem II. Relevant deviation from the norm can be caused by a single application of APAP in higher concentrations used in our experiment. With increasing APAP concentration, there was an increase in carotenoid content. A negative effect of chronic APAP on chlorophyll fluorescence characteristics and primary metabolism was observed. The chronic exposure of xenobiotics has a demonstrably negative effect on the monitored parameters in comparison with the acute action.

## Figures and Tables

**Table 1:** Concentration schema.

Control	Control
5 µM acute	5 µM chronic
50 µM acute	50 µM chronic
500 µM acute	500 µM chronic
5 mM acute	5 mM chronic

**Table 2:** Content of photosynthetically active pigments (nM/cm^2^); arithmetic mean ± standard deviation.

		Suma of Chlorophyll	Carotenoids
5 µM	acute	3.498 ± 0.334	0.643 ± 0.067
	chronic	3.543 ± 0.317	0.669 ± 0.063
50 µM	acute	3.439 ± 0.343 *	0.667 ± 0.063
	chronic	3.849 ± 0.308	0.739 ± 0.057 *
500 µM	acute	3.665 ± 0.320	0.692 ± 0.061
	chronic	4.455 ± 0.320 *	0.824 ± 0.059 *
5 mM	acute	3.779 ± 0.375	0.706 ± 0.067 *
	chronic	4.057 ± 0.338 *	0.790 ± 0.061 *
Control	acute	3.495 ± 0.367 *	0.581 ± 0.073 *
	chronic	3.495 ± 0.367 *	0.581 ± 0.073 *

* Indicates significant differences (*p* < 0.05) compared to the control variant; ns = not significant.

**Table 3:** Maximum quantum efficiency of photosystem II (F_v_/F_m_) (arithmetic mean ± standard deviation).

		F_v_/F_m_	F_v_/F_0_	F_m_/F_0_
5 µM	acute	0.788 ± 0.025 *	4.508 ± 0.542 *	5.747 ± 0.670 *
	chronic	0.783 ± 0.030 *	4.079 ± 0.599 *	5.208 ± 0.667 *
50 µM	acute	0,786 ± 0.029 *	4.182 ± 0.599 *	5.197 ± 0.649 *
	chronic	0.779 ± 0.033 *	3.970 ± 0.684 *	5.397 ± 0.748 *
500 µM	acute	0.776 ± 0.036 *	4.369 ± 0.558 *	5.208 ± 0.661 *
	chronic	0.769 ± 0.039 *	4.060 ± 0.686 *	5.553 ± 0.649 *
5 mM	acute	0.765 ± 0.041 *	4.179 ± 0.588 *	5.458 ± 0.665 *
	chronic	0.745 ± 0.057 *	3.921 ± 0.655 *	5.266 ± 0.676 *
control	acute	0.826 ± 0.015	4.814 ± 0.452	5.815 ± 0.452
	chronic	0.826 ± 0.015	4.814 ± 0.452	5.815 ± 0.452

* Indicates significant differences (*p* < 0.05) compared to the control variant; ns = not significant.

## Abbreviations

APAP—acetaminophen; ATP—adenosine triphosphate; F_v_/F_m_—maximal efficiency of PSII; F_v_/F_0_—maximum primary yield of PSII; Chl—chlorophyll; Car—carotenoids; NPQ—non-photochemical quenching; PAR—photosynthetically active radiation; PSII—photosystem II; SDV—sterilized distilled water.
